# Associations between antibiotic prescriptions and recurrent urinary tract infections in female college students

**DOI:** 10.1017/S0950268818003369

**Published:** 2019-03-08

**Authors:** S. N. Rich, E. M. Klann, C. R. Almond, E. M. Larkin, G. Nicolette, J. D. Ball

**Affiliations:** 1 Department of Epidemiology, College of Public Health and Health Professions, College of Medicine, University of Florida, Gainesville, FL, USA; 2 Department of Neurology, College of Medicine, University of Florida, Gainesville, FL, USA; 3 Department of Agricultural and Biological Engineering, College of Agriculture and Life Sciences, University of Florida, Gainesville, FL, USA; 4 Student Health Care Center, University of Florida, Gainesville, FL, USA; 5 Department of Community Health and Family Medicine, College of Medicine, University of Florida, Gainesville, FL, USA; 6 Emerging Pathogens Institute, University of Florida, Gainesville, FL, USA

**Keywords:** Antibiotics, bacterial infections, epidemiology, urinary tract infections (UTIs)

## Abstract

Urinary tract infections (UTIs) are common among college-aged women and often recur. Some antibiotics recommended to treat UTIs trigger dysbiosis of intestinal and vaginal microbiomes – where uropathogens originate, though few studies have investigated associations between these therapies with recurrent infections. We retrospectively analysed the electronic medical records of 6651 college-aged women diagnosed with a UTI at a US university student health centre between 2006 and 2014. Women were followed for 6 months for incidence of a recurrent infection. In a secondary analysis, associations in women whose experienced UTI recurrence within 2 weeks were also considered for potential infection relapse. Logistic regression was used to assess associations between infection recurrence or relapse and antibiotics prescribed, in addition to baseline patient characteristics including age, race/ethnicity, region of origin, year of encounter, presence of symptomology, pyelonephritis, vaginal coinfection and birth control consultation. There were 1051 instances of infection recurrence among the 6620 patients, indicating a prevalence of 16%. In the analysis of patient characteristics, Asian women were statistically more likely to experience infection recurrence whereas African American were less likely. No significant associations were identified between the antibiotic administered at the initial infection and the risk of infection recurrence after multivariable adjustment. Treatment with trimethoprim-sulphamethoxazole and being born outside of the USA were significantly associated with increased odds of infection relapse in the multivariate analysis. The results of the analyses suggest that treatment with trimethoprim-sulphamethoxazole may lead to an increased risk of UTI relapse, warranting further study.

## Introduction

Community associated urinary tract infections (UTIs) are among the most common indications for outpatient antibiotic prescriptions in the United States [1]. For women, the lifetime prevalence of a UTI is over 50% [2], with nearly one in every three requiring antibiotic therapy for a UTI by age 24 [3]. For some women, UTI symptoms are not resolved immediately with antibiotic therapy in the first 2 weeks, leading to additional alternative antibiotic prescriptions to treat the relapsing infection [2]. Similarly, UTI recurrence – defined as a second, unrelated episode of UTI occurring within 6 months of the initial infection – is also a possible sequelae [2]. Recurrent infections are estimated to affect 30%–44% of women who experience UTIs [4] and can lead to increased annual sick days, healthcare visits, antibiotic prescriptions and days with limited activity [5]. In the USA, women between the ages of 18 and 34 years have the second highest incidence of recurrent UTI, second only to women ages 55–66 years [2]. UTI recurrence is particularly high among sexually active college women [6], among whom additional risk factors include a new sexual partner, frequency of intercourse and spermicide use [7].

Other modifiable clinical factors that could potentially influence the risk of UTI recurrence, such as the choice of empiric antibiotic therapy, are not as well characterised. UTIs are often inappropriately treated [8] and it is unknown whether this is related to infection recurrence. The current national guidelines recommend the use of nitrofurantoin or trimethoprim-sulphamethoxazole as first-line treatment options, and to restrict the use of fluoroquinolones (e.g. ciprofloxacin or norfloxacin) to second-line options [9]. Treatment of UTIs with some of these antibiotics is believed to trigger alterations of both the intestinal microbiota where most uropathogens originate [10], and the vaginal microbiota from where probable uropathogens begin their ascension into the urinary tract [11]. Treatment of UTIs with trimethoprim-sulphamethoxazole has been linked to increased uropathogen resistance against trimethoprim-sulphamethoxazole among college students [12, 13], with one study reporting an increased likelihood of clinical treatment failure for women treated with this antibiotic [12]. Treatment with fluoroquinolones, which are the most commonly prescribed antibiotic category for this condition in the USA [14], has been linked with increased colonisation of the intestinal microbiota with antibiotic-resistant *Enterobacteriaceae* [15]. Further, women who experience recurrent UTIs have been observed to have decreased vaginal colonisation of protective lactobacilli species, relative to women without recurrent UTIs [11]. Some women who present with symptoms of UTI (dysuria, urgency, frequency) are simultaneously experiencing vaginitis or vulvovaginitis at the time of their encounter [16]. The temporality of genitourinary coinfections is somewhat obscured, however, as the development of both UTIs and vaginitis or vulvovaginitis share common risk factors such as impaired immune functions and uncontrolled diabetes [17, 18].

In this study, we sought to understand the relationship between empiric antibiotic prescriptions for uncomplicated UTIs and the likelihood of experiencing a recurrent infection while also considering vaginal coinfections, as well as individual patient and clinical characteristics. The primary objectives were to: (1) estimate the prevalence of UTI recurrence among women diagnosed with an initial episode during the study period, (2) describe the differences in baseline characteristics of patients with sporadic *vs.* recurrent UTI and (3) assess associations between antibiotic therapies prescribed for the initial UTI and the prevalence of recurrent UTI. We hypothesised that women who received empiric prescription of antibiotics associated with higher rates of antimicrobial resistance (trimethoprim-sulphamethoxazole and fluoroquinolones) were more likely to experience recurrent episodes of UTI. As a secondary objective, we also sought to examine the associations between empiric antibiotic therapy and UTI recurrence within 2 weeks, which we considered as potential infection relapse.

## Methodology

### Study design

This was a retrospective cohort study which analysed electronic medical records data for female college students diagnosed with UTIs at a US university student health centre between 2006 and 2014. Women aged 18–34 years who presented at the Student Health Care Center (SHCC) with newly onset UTIs, defined as the earliest clinical encounter assigned an International Classification of Diseases-Ninth Revision (ICD-9) diagnostic code of: ‘599.0’ – ‘Urinary tract infection, unspecified’ or ‘595.*’ – ‘Cystitis’ were included in the analysis.

### Exclusion criteria

Women were excluded from the study population if they had evidence of a potentially complicated UTI, for instance, if they presented with comorbid diabetes (ICD-9: 250.* – ‘Diabetes mellitus’), obesity (ICD-9: 278.00 – ‘Obesity, unspecified’), immunosuppression (ICD-9: 279.* – ‘Disorders involving the immune system’), kidney stones (ICD-9: 592.0 – ‘Calculus of kidney’ and 594.* – ‘Calculus of lower urinary tract’), or a urinary tract abnormality (ICD-9: 599.8 – ‘Unspecified disorder of urethra and urinary tract’, 593.* – ‘Other disorders of kidney and ureter’, 596.4 – ‘Atony of bladder’, 596.5 – ‘Other functional disorders of bladder’). Additionally, women who were pregnant at the time of the first encounter (ICD-9: V22.* – ‘Normal pregnancy’) or who had a history of prior UTIs (ICD-9: V13.02 – ‘Personal history, urinary (tract) infection’) were also excluded from the study. To prevent potential exposure misclassification, women who were prescribed more than two antibiotics at the time of the initial encounter were also excluded.

### Recurrent urinary tract infection

Women were classified as having a recurrent UTI if they developed a second episode of UTI (ICD-9: ‘599.0’ – ‘Urinary tract infection, unspecified’ or ‘595.*’ – ‘Cystitis’) within 6 months of the initial infection. A cut-off date of 30 June 2014 was imposed for initial UTIs to allow adequate follow-up time for secondary infections to develop. Observations not meeting the criteria for recurrent UTI were considered as controls or women with ‘sporadic UTI’. In the secondary analysis, ‘UTI relapse’ was considered in women whose recurrent UTI occurred within 2 weeks of the initial infection.

### Antibiotic exposure

Antibiotic exposures were determined using the available medication information associated with the initial UTI encounter from the electronic medical records. The antibiotics were categorised into the following categories: fluoroquinolones (e.g. ciprofloxacin, levofloxacin), nitrofurantoin (e.g. Macrobid, Macrodantin), trimethoprim/trimethoprim-sulphamethoxazole (e.g. Septa DS, Bactrim, Sulfatrim), a combination of two antibiotics, or other (macrolides (e.g. Zithromax, azithromycin, erythromycin), beta-lactams (amoxicillin, penicillin, augmentin), tetracyclines, cephalosporins, fosfomycin and azoles). Antibiotic prescriptions were categorised regardless of the dose amount listed. Women who were prescribed a two-antibiotic-combination served as the reference group in multivariable analysis.

### Covariates

The data on age (18–20, 21–23 or 24–34 years), race/ethnicity (Caucasian, African American, Hispanic/Latino, Asian, other or unknown), region of origin (USA, outside of the USA or unknown) and year of initial encounter (2006–2014) were obtained from the University Registrar. The data on patients’ clinical encounters, including symptom presentation and comorbidities were extracted from electronic medical records using ICD-9 codes. Variables of interest included those related to UTI symptomology [dysuria (ICD-9: 788.1 – ‘Dysuria’), frequency (ICD-9: 788.41 – ‘Urinary frequency’), urgency (ICD-9: 788.63 – ‘Urgency of urination’), haematuria (ICD-9: 599.7 – ‘Hematuria’) and other (ICD-9: 788.* – ‘Symptoms involving urinary system’)], presence of pyelonephritis (ICD-9: 590.* – ‘Infections of kidney’), presence of vaginal coinfection (ICD-9: 616.1 – ‘Vaginitis and vulvovaginitis’ or 112.1 – ‘Candidiasis of vulva and vagina’) and birth control consultation (ICD-9: V25.* – ‘Encounter for contraceptive management’).

### Statistical analysis

The prevalence of recurrent UTI was calculated as the proportion of women diagnosed with at least one UTI who experienced a recurrent infection within 6 months of the initial infection over the study period. Characteristics of women who experienced sporadic episodes and women who experienced recurrent episodes were compared using *χ*^2^ test for independence. Univariate and multiple logistic regression were performed to predict the binary outcome of recurrent UTI as a function of the type of antibiotic prescribed at the initial infection and on other baseline patient characteristics: age category (reference of 24–34 years), race/ethnicity (reference of Caucasian), region of origin (reference of USA) and vaginal coinfection (reference of ‘no’). Univariate and multiple logistic regression analyses were then used to predict the binary outcome of relapsing UTI in the secondary analysis. All analyses were conducted using R statistical software.

## Results

### Characteristics of study population

**Fig. 1.**
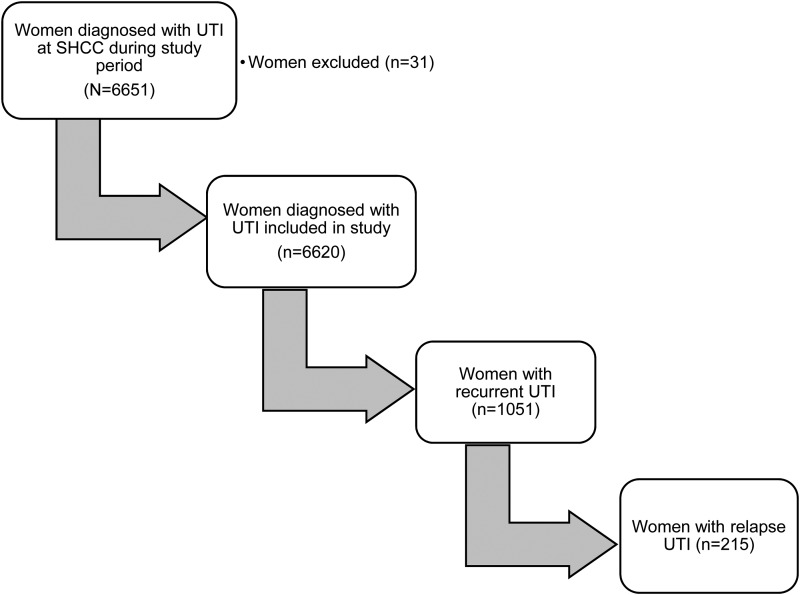
Study population.

Between 2006 and 2014, 6651 women were diagnosed with a UTI at the SHCC. Among those included in the study (*n* = 6620, Fig. 1), 1051 (15.9%) developed a recurrent UTI and 215 (3.2%) experienced relapse. In the *χ*^2^ analysis of baseline patient characteristics, when compared with women with sporadic UTI, women with recurrent UTI were significantly more likely to be between the ages of 18 and 20 years (44.0% *vs.* 49.3%, *P* < 0.01), significantly less likely to be Caucasian (61.3% *vs.* 59.1%, *P* < 0.01) or African American (7.5% *vs.* 5.0%, *P* < 0.01), and significantly more likely to be from the USA (67.4% *vs.* 71.7%, *P* < 0.01) (Table 1). Women with recurrent UTI were also significantly less likely to be diagnosed with a vaginal coinfection at the initial encounter (2.4% *vs.* 3.7%, *P* = 0.04) and significantly more likely to be prescribed a trimethoprim-sulphamethoxazole antibiotic (62.2% *vs.* 58.1%, *P* = 0.02) compared with women with sporadic UTI (Table 1). Compared with women with sporadic UTI, women with recurrent UTI did not differ significantly with respect to the year of encounter, the presence of pyelonephritis, urinary tract symptoms or birth control consultation. Women who experienced UTI relapse were significantly more likely to be prescribed trimethoprim-sulphamethoxazole (73.0% *vs.* 58.1%, *P* < 0.01) and to be from a region outside of the USA (10.2% *vs.* 4.8%, *P* < 0.01) compared with women with sporadic UTI (Table 1).

**Table 1.** Comparison of characteristics of women with sporadic, recurrent and relapse UTI, 2006–2014

Characteristic	Sporadic UTI (*n* = 5569)	Recurrent UTI^a^ (*n* = 1051)	*P*-value*	Relapse UTI (*n* = 215)	*P*-value**
Frequency (%)					
Age (years)			0.007		0.569
18–20	2452 (44.0)	518 (49.3)		88 (40.9)	
21–23	2201 (39.5)	378 (36.0)		87 (40.5)	
24–34	916 (16.4)	155 (14.7)		40 (18.6)	
Race/ethnicity			0.006		0.070
Caucasian	3416 (61.3)	621 (59.1)		125 (58.1)	
Asian	426 (7.6)	103 (9.8)		18 (8.4)	
African American	419 (7.5)	53 (5.0)		8 (3.7)	
Hispanic/Latino	929 (16.7)	192 (18.3)		42 (19.5)	
Other	240 (4.3)	50 (4.8)		16 (7.4)	
Unknown	139 (2.5)	32 (3.0)		6 (2.8)	
Birthplace			0.006		0.001
USA	3753 (67.4)	754 (71.7)		146 (67.9)	
Outside USA	269 (4.8)	55 (5.2)		22 (10.2)	
Unknown	1547 (27.8)	242 (23.0)		47 (21.9)	
Year of initial encounter^b^			0.310		0.098
2006	667 (12.0)	121 (11.5)		21 (9.8)	
2007	759 (13.6)	152 (14.5)		30 (14.0)	
2008	733 (13.2)	141 (13.4)		22 (10.2)	
2009	686 (12.3)	131 (12.5)		29 (13.5)	
2010	581 (10.4)	89 (8.5)		14 (6.5)	
2011	582 (10.5)	105 (10.0)		29 (13.5)	
2012	595 (10.7)	126 (12.0)		23 (10.7)	
2013	642 (11.5)	137 (13.0)		37 (17.2)	
2014	324 (5.8)	49 (4.7)		10 (4.7)	
Initial antibiotic			0.020		<0.001
Combination	542 (9.7)	87 (8.3)		12 (5.6)	
Fluoroquinolone	1362 (24.5)	245 (23.3)		37 (17.2)	
Nitrofurantoin	387 (6.9)	64 (6.1)		9 (4.2)	
TMP/SMX	3237 (58.1)	654 (62.2)		157 (73.0)	
Other^c^	41 (0.7)	1 (0.1)			
Symptom			0.823		0.241
Dysuria	1187 (21.3)	224 (21.3)		38 (17.7)	
Other (frequency, urgency, haematuria)	884 (15.9)	159 (15.1)		30 (14.0)	
None	3498 (62.8)	668 (63.6)		147 (68.4)	
Pyelonephritis			0.942		0.366^d^
No	5489 (98.6)	1035 (98.5)		214 (99.5)	
Yes	80 (1.4)	16 (1.5)		1 (0.5)	
Vaginal coinfection			0.041		0.060
No	5363 (96.3)	1026 (97.6)		213 (99.1)	
Yes	206 (3.7)	25 (2.4)		2 (0.9)	
Birth control			0.354		0.415
No	5337 (95.8)	1000 (95.1)		203 (94.4)	
Yes	232 (4.2)	51 (4.9)		12 (5.6)	

TMP/SMX, trimethoprim/sulphamethoxazole.

a Includes women in relapse population.

b Counts reflect cutoff date imposed for initial cases in 2014 (30 June 2014).

c Patients prescribed ‘Other’ antibiotics were removed from the relapsing UTI analysis due to cell sizes <5.

d Fisher's exact test *P*-value.

*P-value for *χ*^2^ comparison between sporadic and recurrent UTI cases.

**P-value for *χ*^2^ comparison between sporadic and relapse UTI cases.

### Associations of patient characteristics and antibiotics with UTI recurrence

In univariate logistic regression analysis of the associations between patient characteristics and recurrent UTI, women aged 18–20 years had 1.3 times increased odds of developing a recurrent UTI as compared with women aged 24–34 years [odds ratio (OR) 1.25, 95% confidence interval (CI) 1.03–1.52] (Table 2). Compared with Caucasian women, Asian women had greater odds of recurrent UTI (OR 1.33, 95% CI 1.05–1.67), whereas African American women had decreased odds of recurrent UTI (OR 0.70, 95% CI 0.51–0.93) (Table 2). Women who were treated for a vaginal infection at the time of the initial encounter had 37% reduced odds of recurrent UTI (OR 0.63, 95% CI 0.41–0.95) (Table 2). There was no association between region of origin or initial antibiotic and recurrent UTI. In the multiple logistic regression analysis, the associations between race/ethnicity and recurrent UTI remained significant, with Asian women having 1.3 times greater odds (OR 1.30, 95% CI 1.02–1.63) and African American women having 31% decreased odds of recurrent UTI (OR 0.69, 95% CI 0.51–0.92) compared with Caucasian women (Table 2). However, associations between age and vaginal coinfection with UTI recurrence did not remain significant after controlling for other confounders.

**Table 2.** Associations of antibiotics and patient characteristics with UTI recurrence, 2006–2014

Characteristic	Unadjusted OR (95% CI)	Adjusted OR (95% CI)
Age (years)		
18–20	1.25 (1.03–1.52)	1.21 (0.99–1.50)
21–23	1.01 (0.83–1.25)	1.03 (0.83–1.27)
24–34	Ref	Ref
Race/ethnicity		
Caucasian	Ref	Ref
Asian	1.33 (1.05–1.67)	1.30 (1.02–1.63)
African American	0.70 (0.51–0.93)	0.69 (0.51–0.92)
Hispanic/Latino	1.14 (0.95–1.36)	1.12 (0.93–1.33)
Other	1.15 (0.83–1.56)	1.31 (0.90–1.88)
Unknown	1.27 (0.84–1.85)	1.37 (0.91–2.01)
Birthplace		
USA	Ref	Ref
Outside USA	1.02 (0.75–1.36)	0.92 (0.65–1.28)
Initial antibiotic		
Combination	Ref	Ref
Fluoroquinolone	1.12 (0.86–1.47)	1.00 (0.76–1.33)
Nitrofurantoin	1.03 (0.73–1.46)	0.91 (0.63–1.30)
TMP/SMX	1.26 (0.99–1.61)	1.11 (0.86–1.45)
Other	0.15 (0.01–0.71)	0.15 (0.01–0.69)
Vaginal coinfection		
No	Ref	Ref
Yes	0.63 (0.41–0.95)	0.72 (0.44–1.11)

TMP/SMX, trimethoprim/sulphamethoxazole; OR, odds ratio; CI, confidence interval; ref, reference.

### Associations of patient characteristics and antibiotics with UTI relapse

In the multivariate analysis of the associations between antibiotics and patient characteristics with UTI relapse, women who were born outside of the USA had 2.1 times increased odds of UTI relapse compared with women born in the USA (adjusted OR 2.12, 95% CI 1.30–3.31) (Table 3). Women treated with trimethoprim-sulphamethoxazole had 2.1 times increased odds of UTI relapse compared with women treated with combination therapy (adjusted OR 2.14, 95% CI 1.23–4.09) (Table 3).

**Table 3.** Associations of antibiotics and patient characteristics with UTI relapse, 2006–2014

Characteristic	Unadjusted OR (95% CI)	Adjusted OR (95% CI)
Birthplace		
USA	Ref	Ref
Outside USA	2.10 (1.29–3.28)	2.12 (1.30–3.31)
Initial antibiotic		
Combination	Ref	Ref
Fluoroquinolone	1.23 (0.65–2.47)	1.21 (0.64–2.43)
Nitrofurantoin	1.05 (0.42–2.51)	1.01 (0.41–2.40)
TMP/SMX	2.19 (1.26–4.19)	2.14 (1.23–4.09)

TMP/SMX, trimethoprim/sulphamethoxazole; OR, odds ratio; CI, confidence interval; ref, reference.

## Discussion

The objectives of this study were to estimate the prevalence of recurrence among women diagnosed with a UTI, compare the characteristics of patients with recurrent *vs.* sporadic UTI and assess associations between antibiotic prescriptions and patient characteristics with recurrent UTI in this population of female college students. As a secondary objective, we also considered the associations between antibiotic prescriptions and patient characteristics with UTI relapse in women whose recurrent infection occurred within 2 weeks of the initial infection. The prevalence of UTI recurrence and relapse in this population was 16% and 3%, respectively. The proportion of women with recurrent UTI is comparable with a previous study conducted in a similar population of female college students which reported a prevalence of 19% [19], however it is much lower than other estimates. The American Urogynecologic Society estimates recurrence occurs in 30% to 44% of all women who experience a UTI [7]. Considering the potential for women in this study to have presented with symptoms of recurrent UTI at other health centres, it is possible the prevalence of recurrent UTI was underestimated in this study.

No significant associations were identified between the antibiotic administered at the initial infection and the risk of infection recurrence after multivariable adjustment. Treatment with trimethoprim-sulphamethoxazole was significantly associated with increased odds of infection relapse in the multivariate analysis, however, which suggests treatment with this antibiotic may have a marginal influence on the risk of UTI recurrence in some women. Contrary to our hypothesis, fluoroquinolones were not significantly associated with recurrent UTI in this study.

The only characteristic significantly associated with recurrent UTI after multivariable adjustment was race/ethnicity. When compared with Caucasian women, Asian women had increased odds of recurrent UTI whereas African American women had decreased odds. The reasons for these findings are unclear, though one previous study conducted in another population of college women similarly found an increased frequency of recurrent UTI in Asian women [20]. A study conducted among women with non-recurrent UTI in Philadelphia found Asian women had significantly increased odds of infection with a uropathogen possessing lower susceptibility to fluoroquinolones by a factor of 3 [21]. Given that resistant uropathogens are associated with recurrent infections [19], this could potentially explain the higher prevalence observed among Asian women in this study, though fluoroquinolone use was not significantly associated with recurrent UTI in this analysis. For African American females, a previous meta-analysis reported a significantly lower risk of UTI in paediatric populations [22], though the biological mechanism through which this occurs remains unclear. In a study investigating the health perceptions of individuals with lower urinary tract symptoms, African American respondents more often attributed their symptoms to personal behaviours and decided not to seek medical care [23]. This could potentially explain the reduced odds of recurrent infection observed in this population, however, the differences could also be due to health behaviours or genetic susceptibilities which were not assessed. In the univariate analysis, women prescribed trimethoprim-sulphamethoxazole had increased odds of recurrent UTI when compared with women prescribed combination therapy.

Considering other factors, age and the presence of a vaginal coinfection were found to be associated with recurrent UTI during the univariate analysis. Women in the youngest age group (18–20 years) were found to have increased odds of recurrent UTI when compared with women in the older age group (24–34 years). This differs from previous studies conducted among college women, with one study finding no association between UTI recurrence and age [20] and another which reported a higher rate ratio for women in the oldest age group [19]. Vaginal coinfection was associated with decreased odds of both UTI recurrence and UTI relapse in the univariate analyses, suggesting a protective effect of having this simultaneous diagnosis. Women diagnosed with both infections may have been treated with two antibiotics (i.e. combination therapy), which explains the lack of a significant association observed after multivariable adjustment.

To our knowledge, this was the first study to identify factors associated with UTI relapse (infection recurrence within 2 weeks) in female college students. UTI relapse was significantly associated with being born outside of the USA and being prescribed trimethoprim-sulphamethoxazole. The association of UTI relapse with trimethoprim-sulphamethoxazole may be due to increased rates of resistance, which has been previously been observed in a population of college students [13].

Among the strengths of this study included the large sample size of 6651 women between the ages of 18 and 34 years which is the second highest incident age group for recurrent UTI [2]. Additionally, the span of time of nine consecutive years and the diversity of the population with respect to race/ethnicity and region of origin were also advantageous. Another strength of the study was the uniform method of data extraction and collection as well as the singular source of electronic medical records used throughout this study period.

The study also had limitations that warrant discussion. First, this study assumed that the first UTI diagnosed at the SHCC is the true initial UTI and that women who experienced a secondary infection presented at the SHCC. However, it's possible that women could have presented with symptoms of UTI at other health centres before, during or after they presented at the SHCC. Considering this, the prevalence of UTI recurrence may be underestimated in this population as women may be seeking treatment for secondary infections at different health centres. This may explain the relatively low prevalence of recurrence observed in this population. Second, we were not able to distinguish between a true relapse (infection with the same organism) and recurrent infection (infection likely due to an organism that differs from the initial infection) due to a lack of available information regarding the species of each infection, though our definitions of both infections are consistent with previous studies [7, 19]. Further, some important risk factors could not be measured or accounted for due to the lack of availability within the electronic medical records, including exposure to any other antibiotics not related to UTI treatment within the past 3 months, adherence to antibiotic regimens, drug allergies, sexual and hygienic behaviours and other lifestyle factors.

## Conclusion and future directions

In conclusion, this study investigated factors associated with UTI recurrence in a higher risk group of female college students. No associations were identified between the antibiotic administered at the initial infection and the risk of recurrent UTI after multivariable adjustment. However, treatment with trimethoprim-sulphamethoxazole was significantly associated with increased risk of UTI relapse. Future studies should be conducted to estimate the prevalence of trimethoprim-sulphamethoxazole resistance in this region, as use of this antibiotic as a first-line option should be reconsidered when local resistance to *Escherichia coli* exceeds 20% [7]. Although fluoroquinolones are not recommended as first-line agents for uncomplicated UTI due to increasing resistance and potentially serious adverse side effects [24], there was no evidence to suggest that use of this antibiotic influenced the risk of recurrent UTI in this study. Fluoroquinolones were the second most commonly prescribed antibiotic in this study population (after trimethoprim-sulphamethoxazole) and therefore effort to reduce prescribing of this antibiotic at the SHCC should be encouraged. More research is needed to elucidate the mechanisms by which race/ethnicity and region of origin contribute to risk of UTI recurrence by considering health behaviours, such as treatment adherence, hygiene and sexual activity, in addition to uropathogen characteristics.
